# Potential Interaction between *WNT16* and Vitamin D on Bone Qualities in Adolescent Idiopathic Scoliosis Patients and Healthy Controls

**DOI:** 10.3390/biomedicines12010250

**Published:** 2024-01-22

**Authors:** Guangpu (Kenneth) Yang, Huanxiong Chen, Ka-Lo Cheng, Man-Fung Tang, Yujia Wang, Lik-Hang (Alec) Hung, Chun-Yiu (Jack) Cheng, King-Lun (Kingston) Mak, Yuk-Wai (Wayne) Lee

**Affiliations:** 1 SH Ho Scoliosis Research Laboratory, Joint Scoliosis Research Centre of the Chinese University of Hong Kong and Nanjing University, The Chinese University of Hong Kong, Hong Kong, China; 2 Department of Orthopaedics and Traumatology, The Chinese University of Hong Kong, Hong Kong, China; 3 Department of Spine Surgery, The First Affiliated Hospital of Hainan Medical University, Haikou 571199, China; 4 Department of Paediatrics, The Chinese University of Hong Kong, Hong Kong, China; 5 Department of Orthopaedics and Traumatology, Prince of Wales Hospital, Hong Kong, China; 6 Department of Biomedical Sciences, City University of Hong Kong, Hong Kong, China; 7 Li Ka Shing Institute of Health Sciences, The Chinese University of Hong Kong, Hong Kong, China

**Keywords:** adolescent idiopathic scoliosis, WNT16, vitamin D binding protein, vitamin D supplementation

## Abstract

Adolescent idiopathic scoliosis (AIS) is a three-dimensional spinal deformity that is associated with low bone mineral density (BMD). Vitamin D (Vit-D) supplementation has been suggested to improve BMD in AIS, and its outcomes may be related to genetic factors. The present study aimed to (a) investigate the synergistic effect between a low BMD-related gene (wingless-related integration site 16, *WNT16*) and two important Vit-D pathway genes (Vit-D receptor, *VDR,* and Vit-D binding protein, *VDBP*) on serum Vit-D and bone qualities in Chinese AIS patients and healthy adolescents, and (b) to further investigate the effect of ablating *Wnt16* on the cortical bone quality and whether diets with different dosages of Vit-D would further influence bone quality during the rapid growth phase in mice in the absence of *Wnt16*. A total of 519 girls (318 AIS vs. 201 controls) were recruited, and three selected single-nucleotide polymorphisms (SNPs) (*WNT16* rs3801387, *VDBP* rs2282679, and *VDR* rs2228570) were genotyped. The serum 25(OH)Vit-D level was significantly associated with *VDBP* rs2282679 alleles (OR = −4.844; 95% CI, −7.521 to −2.167, *p* < 0.001). Significant multi-locus models were identified by generalized multifactor dimensionality reduction (GMDR) analyses on the serum 25(OH)Vit-D level (*p* = 0.006) and trabecular area (*p* = 0.044). In the gene-edited animal study, *Wnt16* global knockout (KO) and wildtype (WT) male mice were provided with different Vit-D diets (control chow (1000 IU/Kg) vs. Vit-D-deficient chow (Nil in Vit-D) vs. high-dose Vit-D chow (20,000 IU/Kg)) from 4 weeks to 10 weeks old. *Wnt16* global KO mice had significantly lower serum 25(OH)Vit-D levels and higher liver *Vdbp* mRNA expression levels than WT mice. In addition, *Wnt16* global KO mice showed a decrease in bone density, cortical thickness and cortical area compared with WT mice. Interestingly, high-dose Vit-D chow led to lower bone density, cortical thickness, and cortical area in WT mice, which were less obvious in *Wnt16* global KO mice. In conclusion, *WNT16* may regulate the serum 25(OH)Vit-D level and bone qualities, which might be associated with *VDBP* expression. Further investigations with a larger sample size and wider spectrum of scoliosis severity are required to validate our findings regarding the interaction between *WNT16* and Vit-D status in patients with AIS.

## 1. Introduction

Adolescent idiopathic scoliosis (AIS) is a three-dimensional spinal deformity with a global prevalence from 1% to 4% [1]. The scoliotic curve is stable in the majority of AIS patients, but if these curve progress, it could cause severe health-related problems including disability and reduced quality of life [2]. The etiopathogenesis of AIS is largely un-known. It is generally believed that AIS is a multifactorial disease in which the onset and progression are regulated by various genetic and non-genetic factors to different extents [1]. A better understanding of the etiopathogenesis of AIS will reshape the prognoses and treatment approaches.

Approximately 38% of AIS patients were shown to have a low bone mineral density (BMD), which was defined by a BMD Z-score < −1 based on Dual-Energy X-ray Absorptiometry (DXA). The low BMD in AIS appears to be systemic [3,4,5] and, if untreated, it could persist until skeletal maturity [6,7]. Recent studies using high-resolution peripheral quantitative computed tomography (HR-pQCT) have revealed disturbed bone qualities in both cortical and trabecular compartments in the distal radius [8,9]. The low BMD was shown to possess significant prognostic value for curve progression in AIS [10,11]. Furthermore, the iliac bone biopsies from AIS patients showed higher trabecular separation, a lower trabecular connectivity density, and a lower calcium-to-carbon ratio than non-AIS controls [12]. Osteocyte lacuna-canalicular networks were found to have an abnormal morphology and were dysfunctional in AIS [13].

Considering the close association between low BMD and curve progression, vitamin D (Vit-D) has been postulated to be a possible strategy to reduce the risk of curve progression through its beneficial effect on bone mass [14,15,16]. The circulating level of 25(OH)Vit-D is commonly used to define Vit-D status, which is correlated with bone quality and fracture risk in children and adolescents [17,18]. Since the effect of Vit-D supplementation on bone density in adolescents is controversial, [19,20] it is suspected that the response to Vit-D supplementation varies among children and adolescents, which may be related to undefined genetic factors. Therefore, a better understanding of the genotypes that could modulate Vit-D metabolism and Vit-D’s effect on bone density will be of clinical interest in optimizing the benefit of Vit-D supplementation for AIS patients.

Various loci or genes have been reported to be associated with low BMD and/or osteoporosis [21,22]. Recent genome-wide association studies (GWAS) revealed a novel locus named wingless-related integration site 16 (*WNT16*) in the WNT signaling pathway—a key biological pathway that regulates bone homeostasis. GWAS data confirmed that *WNT16* variants were associated with cortical bone thickness, BMD, and fracture risk [23,24]. However, previous studies focused on individual single-nucleotide polymorphisms (SNPs) and did not take into account the gene–gene interactions in multifactorial diseases. Whether low BMD-related SNPs interact with those related to the Vit-D pathway to produce a benefit from Vit-D supplementation has not been well documented.

We hypothesized that genetic variance in *WNT16* might affect bone accrual during the rapid growth phase, thus resulting in reduced cortical bone thickness and BMD in AIS. Even though *WNT16* is a potential therapeutic target, there is a lack of understanding on how its biological activity and expression level are modulated. The present study aimed to (a) investigate the interaction between a low BMD-related gene (*WNT16*) and two important Vit-D pathway genes (Vit-D receptor, *VDR*, and Vit-D binding protein, *VDBP*) on serum 25(OH)Vit-D and bone qualities in Chinese AIS patients and healthy adolescents, and (b) to further investigate the effect of ablating *Wnt16* on cortical bone quality and whether diets with different dosages of Vit-D would further influence bone quality during the rapid growth phase in mice in the absence of *Wnt16*.

## 2. Methods

### 2.1. Patient Recruitment and Clinical Assessment

Chinese girls who were diagnosed with AIS by at least two senior orthopedic surgeons through spine radiographs were recruited from the Scoliosis Clinic of The Prince of Wales Hospital with approval from the joint CUHK-NTEC Clinical Research Ethics Committee (reference number: 2017.026). The severity of the scoliotic curve was measured using the Cobb angle following the Cobb method [25]. Patients who had a history of surgery or medicines affecting bone health or measurement of bones were excluded. Healthy Chinese girls whose spine showed no scoliosis or other spine deformities were recruited from a secondary school in Hong Kong to serve as the gender-matched controls. Written informed consent was obtained from all participants and their legal guardians. Anthropometric parameters of all subjects including body weight, standing height, sitting height, and arm span were measured using a standardized method [26]. Pubertal maturity was assessed using the Tanner staging system. The Tanner stage was self-reported by the subjects guided by showing a Tanner stage pictorial essay [27,28].

### 2.2. Bone Qualities and Serum Vit-D Levels of the Clinical Cohort

DXA (Hologic, Horizon DXA System, Marlborough, MA, USA) was used to assess areal BMD (aBMD) in the non-dominant bilateral femoral necks (g/cm^2^) [9]. The age and gender-adjusted Z-score were calculated with the adopted normative aBMD dataset from Chinese girls [10]. Although the AIS is a spinal deformity, the lumber spinal BMD was not used because its value could be affected by the rotated vertebra deformity in AIS [29,30]. The volumetric BMD (vBMD), bone geometry, and bone microstructure in the non-dominant distal radius were assessed by HR-pQCT (Xtreme CT I, Scanco Medical, Bruttisellen, Switzerland) [31]. A reference line was set at the most proximal point of the inner aspect of the growth plate, and 5 mm proximal to the reference line was the starting point for each scan. A 9.02 mm segment spanning our region of interests (ROIs) was adopted and a resolution of 82 μm was used. The bone qualities were measured and calculated according to the manufacturer’s protocol and included the total bone area (mm^2^), cortical bone area (mm^2^), trabecular bone area (mm^2^), cortical perimeter (mm), cortical thickness (mm), trabecular thickness (mm), trabecular number (mm^−1^), total volumetric BMD (vBMD) (mg/mm^3^), cortical vBMD (mg/mm^3^), and trabecular vBMD (mg/mm^3^). Blood samples were taken from the subjects on the same day as the bone measurements and were stored at ultra-low freezers (−80 °C) until further analysis. Collected blood was centrifuged at 12,000 rpm for 10 min at 4 °C. Serum 25(OH)Vit-D level was measured by liquid chromatography isotope-dilution electrospray ionization tandem mass spectrometry (LCTMS) as described previously [32]. Vit-D sufficiency was defined as serum 25(OH)Vit-D > 50 nmol/L, insufficiency as 25 ≤ 25(OH)Vit-D ≤ 50 nmol/L, and deficiency as 25(OH)Vit-D < 25 nmol/L [33,34].

### 2.3. SNP Selection and Genotyping of the Clinical Cohort

Three SNPs that were reported to be associated with BMD or serum 25(OH)Vit-D levels were selected for *VDR*, *VDBP*, and *WNT16*. Genomic DNA was extracted from peripheral blood leucocytes of all subjects and the SNPs were genotyped using the iPLEX assay with primers designed by the Beijing Genomics Institute (BGI, Shenzhen, China). The subjects and the staff performing the clinical assessments were blinded to the genotyping results until the end of the study.

### 2.4. Wnt16 Global Knockout and Vit-D Diets in Mice

The animal study was approved by the Animal Experimentation Ethics Committee (AEEC) at the Chinese University of Hong Kong (CUHK) (reference number: 2017.029). Male *Wnt16* global knockout (KO) mice were generated by disrupting the first three exons in a C57BL6/J-129SvEv hybrid genetic background [35]. Wildtype (WT) mice of homozygous *Wnt16^+/+^* were used as the control. Breeding and genotyping were carried out in accordance with established protocols [36]. Mice were randomly allocated (eight mice in each group) to be fed with one of three semisynthetic diets from 4 weeks old until sacrifice: control chow, Vit-D-deficient chow, or high-dose Vit-D chow. The control chow was standard AIN93G rodent diet (Cat. # SF-AIN93G, Specialty Feeds, Perth, WA, Australia) containing 1000 IU/Kg Vit-D, 0.7% calcium, and 0.35% phosphorous. The Vit-D-deficient chow (Cat. # SF03-009, Specialty Feeds, Perth, WA, Australia) was a diet formulation that contained no Vit-D, 0.47% calcium, and 0.35% phosphorous. High-dose Vit-D chow (Cat. # SF17-207, Specialty Feeds, Perth, WA, Australia) contained 20,000 IU/Kg Vit-D, 0.72% calcium and 0.35% phosphorus. Body weight was measured at 4, 7, and 10 weeks old. For each Vit-D chow, two groups of mice were sacrificed after three weeks (7-week-old) and six weeks (10-week-old) since fed with Vit-D diets.

### 2.5. MicroCT Measurement and Serum Vit-D Level in Mice

Micro-CT (μCT-40, Scanco Medical, Brttisellen, Switzerland) was used to quantify the cortical and trabecular bone quality in the femoral in 7- and 10-week-old mice as we previously described [37,38]. Before the assessment, the mice were sacrificed by cervical dislocation and their right-side femora were collected and fixed with 70% ethanol. An isotropic voxel size of 15 μm with 70 kVp and 114 μA was applied for the scan. The cortical bone analysis was performed in the midshaft femora starting from the inferior border of the great trochanter and extending 50 slices (400 μm) longitudinally. Parameter sigma and support were set to be 0.8 and 1, and the global threshold was set to be 260. The trabecular bone proximal to the distal growth was measured starting at the inferior border of the growth plate and extending a further longitudinal distance of 100 slices (800 μm) with the same parameter sigma and support values as those for cortical bone. The global threshold was set to 200 for trabecular bone. Tissue mineral density (mg/cm^3^), cortical thickness (mm), cortical area (mm^2^), trabecular number (mm^−1^), trabecular thickness (mm), and trabecular separation (mm) were calculated. Serum 25(OH)Vit-D levels were assessed at 10 weeks old. Orbital venous blood was collected with capillary blood collection tubes after general anesthesia using ketamine (90 mg/kg) and xylazine (4.5 mg/kg). The collected blood was subjected to centrifugation at 12,000 rpm for 10 min at 4 °C, and the serum was stored immediately at −80 °C until further analysis. Serum 25(OH)Vit-D levels were measured using an ELISA kit (Cat. # CSB-E08099M, Cusabio Technology LLC, Houston, TX, USA) according to the manufacturer’s instructions.

### 2.6. Messenger Ribonucleic Acid Expression in Mice

The livers, kidneys, and left-side femora were collected immediately after the mice were sacrificed and were freshly frozen in liquid nitrogen. Total RNA from these tissues was isolated using TRNzol reagent (Invitrogen, Carlabad, CA, USA). The RNA was reverse transcribed using a PrimeScript RT Reagent Kit (Cat. #RR036, TaKaRa Bio Inc., CA, USA), and quantitative real time polymerase chain reaction (qRT-PCR) was performed using Power SYBR Green PCR Master Mix (Cat. #4368702, Thermo Fisher Scientific Inc., Waltham, MA, USA) following the manufacturers’ protocols. Beta-actin was employed as the reference gene and the 2^−ΔCt^ method was used in the data analysis. The primers used in this assessment are as follows:

*β-actin:* F: GGCTGTATTCCCCTCCATCG; R: CCAGTTGGTAACAATGCCATGT

*Vdpb:* F: CCTGCTGGCCTTAGCCTTT; R: TGCTCAAATGTGCTACTGGAAA

*Vdr:* F: CACCTGGCTGATCTTGTCAGT; R: CTGGTCATCAGAGGTGAGGTC

### 2.7. Statistical Analysis

For the clinical cohort, the clinical and laboratory characteristics were compared by independent *t*-tests for numerical data and Pearson chi-squared tests for categorical variables. Allele frequencies for all SNPs were estimated by the gene-counting method, and the Hardy–Weinberg equilibrium (HWE) was examined using the χ2 exact test. The associations between SNPs and phenotypes were analyzed by multi-variable regression. All comparisons were two-tailed and conducted using SPSS (version 21, IBM Corp., Armonk, NY, USA). The nominal significance threshold was corrected to 0.0167 using Bonferroni corrections for the numbers of SNPs being genotyped. Generalized multifactor dimensionality reduction (GMDR, version 0.9; http://www.healthsystem.virginia.edu/internet/addiction-genomics/software/ (accessed on 20 December 2020)) was used to test for gene–gene interactions and to pool the multi-locus genotypes into high-risk and low-risk groups following the manufacturer’s protocol. The best model was chosen based on cross-validation consistency (CVC) ≥ 8/10 and a testing accuracy (TA) score ≥ 0.55. Statistical significance was derived empirically from 5000 permutations before selecting the best performing models. For animal studies, independent *t*-tests were used to compare different groups. A *p*-value less than 0.05 was considered to be statistically significant.

## 3. Results

### 3.1. Association between the Individual SNPs and Phenotypes in the Clinical Cohort

In total, 519 girls (318 AIS vs. 201 controls) were recruited, and their baseline age, stage of puberty, anthropometry, vitamin D status, and bone qualities are summarized in Table 1. The body weight was lower and the sitting height was shorter in the AIS group compared to the control group. AIS group had a lower BMD in the femoral neck and distal radius with a disrupted trabecular microarchitecture in the distal radius when compared with the controls. These observations are in line with previous reports on the anthropometric and bone quality characteristics in AIS [1,8,9,39,40]. In the present study, the AIS group were slightly older in age and were in the advanced pubic hair stage. Their serum 25(OH)Vit-D levels were significantly higher than that of the controls.

The genotyping efficiency was ≥98% for the three SNPs and their frequencies followed the principle of the HWE (Supplementary Table S1). The results of the multivariable regression on the associations between each individual SNP and the phenotypes are shown in Supplementary Table S2. In those with the minor allele of rs2282679 in *VDBP*, the serum 25(OH)Vit-D level was significantly lower when compared with the major allele (OR = −4.844; 95% CI, −7.521 to −2.167, *p* < 0.001). There was no correlation between the diagnosis (AIS vs. control) and alleles of the selected SNPs. In this regard, the following gene–gene interaction analyses were performed with a merged cohort of both the AIS and control groups.

### 3.2. Gene–Gene Interactions between WNT16, VDR, and VDBP in the Clinical Cohort

**Figure 1:**
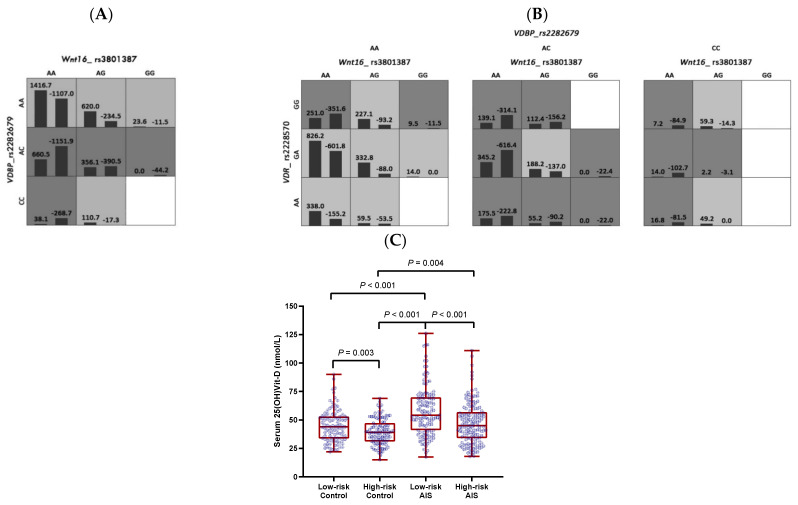
Best twolocus model (**A**) and three-locus model (**B**) for serum 25(OH)VitD level and its distribution in different genotypes (**C**). Highrisk genotypes are in dark grey, lowrisk genotypes are in light grey, and white cells indicate unclassified genotypes due to small numbers of subjects. The figures above the bars in each cell are the sum of scores for the corresponding group of individuals. The heights of the bars are proportional to the sum of scores in each group. Distribution of serum 25(OH)VitD level in each genotype is shown in (**C**) for the threelocus model. The range, quartile, and median are shown in red, and datapoints are shown in blue.

**Figure 2:**
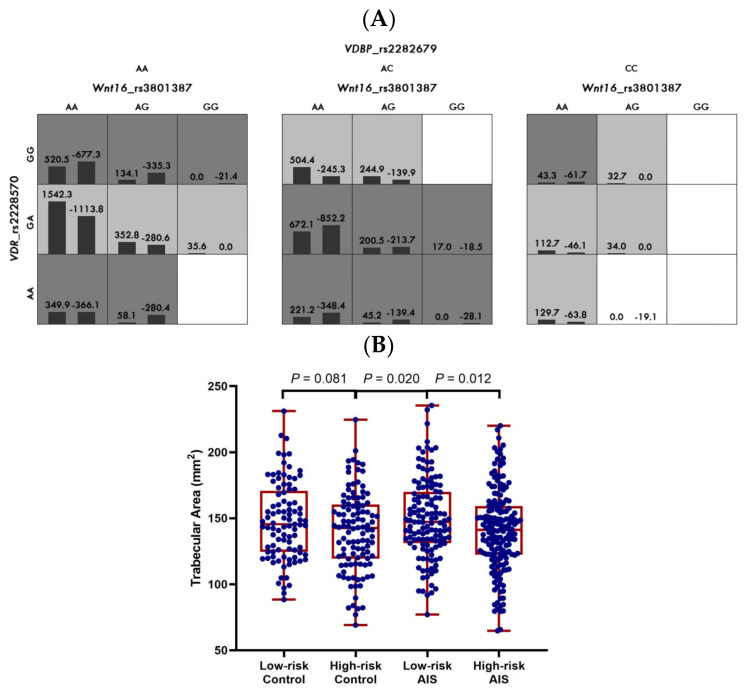
Best threelocus model for trabecular area (**A**) and its distribution in different genotypes (**B**). Highrisk genotypes are in dark grey, lowrisk genotypes are in light grey, and white cells indicate unclassified genotypes due to small numbers of subjects. The figures above the bars in each cell are the sum of scores for the corresponding group of individuals. The heights of the bars are proportional to the sum of scores in each group. Distribution of serum trabecular area in each genotype is shown in (**B**) for the three-locus model. The range, quartile, and median are shown in red, and datapoints are shown in blue.

The significant multi-locus interaction models are summarized in Table 2. For the serum 25(OH)Vit-D level, the GMDR analysis revealed one significant two-locus model formed by *WNT16* rs3801387 and *VDBP* rs2282679 (*p* = 0.003, Figure 1A), and one three-locus model involving all three selected SNPs (*p* = 0.006, Figure 1B). The total serum 25(OH)Vit-D levels were lower among the high-risk group compared to the low-risk group in both the AIS and control groups (Figure 1C). For bone quality parameters, one three-locus model formed by the three selected SNPs was identified (*p* = 0.044, Figure 2A). In this three-locus model, the trabecular area was smaller in the high-risk group than in the low-risk group, which was statistically significant in the AIS group (*p* = 0.012) and marginally significant in the controls (*p* = 0.081, Figure 2B). There were no significant interactive models for the other bone quality parameters.

### 3.3. Serum 25(OH)Vit-D and Vdbp Expression Level in Wnt16 Global Knockout Mice

**Figure 3:**
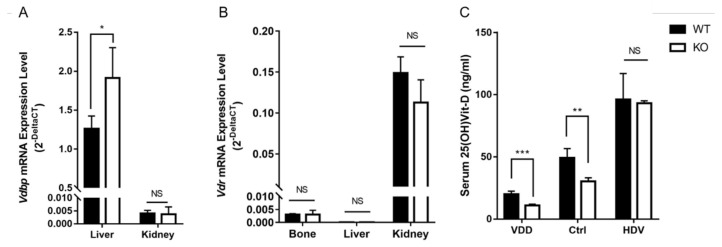
Expression level of *Vdbp* (**A**) and *Vdr* (**B**) mRNA and serum 25(OH)Vit-D level (**C**) in mice. *Vdbp*, vitamin D binding protein; *Vdr*, vitamin D receptor; WT, wildtype mice; KO, *Wnt16* global knockout; VDD, vitamin D deficient chow; Ctrl, control chow; HDV, high-dose vitamin D chow, *, *p* < 0.05; **, *p* < 0.01; ***, *p* < 0.001; NS, not significant.

The body weight of *Wnt16* global knockout (KO) mice was shown to be comparable with that of wildtype mice at the age of 4 weeks (13.3 ± 1.6 g vs. 11.4 ± 3.1 g; *p* = 0.170), 7 weeks (20.7 ± 1.9 g vs. 20.7 ± 1.6 g; *p* = 0.890), and 10 weeks (23.3 ± 0.5 g vs. 23.3 ± 1.0 g; *p* = 0.570). There was a higher level of *Vdbp* mRNA expression in the livers of the *Wnt16* global KO mice than that in the WT mice (1.91 ± 0.38 vs. 1.26 ± 0.16, *n* = 4, *p* = 0.021, Figure 3A). No significant difference in *Vdr* expression between the *Wnt16* global KO mice and WT mice was noted in the bone, liver, or kidney (Figure 3B). The serum 25(OH)Vit-D level was significantly lower in the *Wnt16* global KO mice compared with that in the WT mice in the control chow group (30.4 ± 2.8 ng/mL vs. 49.1 ± 7.6 ng/mL, *n* = 4, *p* = 0.003, Figure 3C) and in the Vit-D-deficient chow group (10.9 ± 1.1 ng/mL vs. 20.0 ± 2.4 ng/mL, *n* = 4, *p* < 0.001). For the mice on the high-dose Vit-D chow, no significant difference was noted between the *Wnt16* global KO mice and WT mice (93.2 ± 1.9 ng/mL vs. 96.2 ± 20.8 ng/mL, *n* = 4, *p* = 0.834).

### 3.4. Bone Qualities in Wnt16 Global Knockout Mice on Different Vit-D Chows

**Figure 4:**
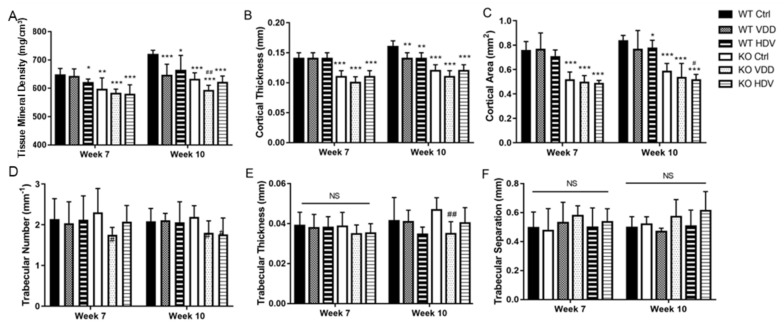
micro-CT analysis on femur of wildtype (WT) and *Wnt16* global knockout (KO) mice at 7-week-old and 10-week-old with either control chow (Ctrl), Vit-D deficient chow (VDD), or high-dose Vit-D chow (HDV). Tissue mineral density (**A**), cortical thickness (**B**), cortical area (**C**), trabecular number (**D**), trabecular thickness (**E**), and trabecular separation (**F**) are shown. *, *p* < 0.05; **, *p* < 0.01 and ***, *p* < 0.001 compared to WT Ctrl group; #, *p* < 0.05 and ##, *p* < 0.01 compared to KO Ctrl group; NS, not significant.

The bone qualities in the mice at 7 and 10 weeks old are summarized in Figure 4. After three weeks on the control chow, the 7-week-old *Wnt16* global KO mice were found to have a lower tissue mineral density (595.38 ± 41.43 mg/cm^3^ vs. 646.17 ± 23.93 mg/cm^3^, *p* = 0.009, Figure 4A), smaller cortical thickness (0.11 ± 0.01 mm vs. 0.14 ± 0.01 mm, *p* < 0.001, Figure 4B), and smaller cortical area (0.52 ± 0.06 mm^2^ vs. 0.76 ± 0.07 mm^2^, *p* < 0.001, Figure 4C) than the WT mice. The difference was more obvious in 10-week-old mice for the tissue mineral density (630.66 ± 23.91 mg/cm^3^ vs. 718.58 ± 15.52 mg/cm^3^, *p* < 0.001), cortical thickness (0.12 ± 0.01 mm vs. 0.16 ± 0.01 mm, *p* < 0.001), and cortical area (0.59 ± 0.06 mm^2^ vs. 0.84 ± 0.07 mm^2^, *p* < 0.001). No significant difference between the *Wnt16* global KO and WT mice was noted for trabecular number, trabecular thickness, or trabecular separation (Figure 4D–F).

After changing to the Vit-D-deficient chow for 6 weeks, the 10-week-old *Wnt16* global KO mice exhibited a significantly lower tissue mineral density (591.09 ± 19.59 mg/cm^3^ vs. 630.66 ± 23.91 mg/cm^3^, *p* = 0.002), trabecular number (1.80 ± 0.26 vs. 2.19 ± 0.25, *p* = 0.034), and trabecular thickness (0.034 ± 0.004 mm vs. 0.038 ± 0.006, *p* = 0.026) than those on the control chow. The 10-week-old WT mice showed similar phenotypes: a lower tissue mineral density (644.68 ± 40.27 mg/cm^3^ vs. 718.58 ± 15.52 mg/cm^3^, *p* = 0.003) and smaller cortical thickness (0.14 ± 0.01 mm vs. 0.16 ± 0.01 mm, *p* = 0.001) than those on the control chow.

A significant decrease in the cortical area was noted in 10-week-old WT mice receiving the high-dose Vit-D chow (0.78 ± 0.06 mm^2^ vs. 0.84 ± 0.04 mm^2^, *p* = 0.033) when compared with the control chow. In addition, consuming the high-dose Vit-D chow led to a lower tissue mineral density (662.40 ± 53.92 mg/cm^3^ vs. 718.58 ± 15.52 mg/cm^3^, *p* = 0.013) and smaller cortical thickness (0.14 ± 0.01 mm vs. 0.16 ± 0.01 mm, *p* = 0.001) in the WT mice at 10 weeks old. In contrast, the 10-week-old *Wnt16* global KO mice had a significantly smaller cortical area (0.49 ± 0.02 mm^2^ vs. 0.59 ± 0.06 mm^2^, *p* < 0.001) and lower trabecular number (1.76 ± 0.37 vs. 2.19 ± 0.25 mm^−1^, *p* = 0.026) when compared with the control chow, while the difference with the control chow was not obvious in tissue mineral density and cortical thickness.

## 4. Discussion

Patients with AIS have been well documented as having a lower bone mass in both the axial and peripheral skeletons. However, the underlying mechanism for bone loss in AIS patients is still uncertain. In the past decade, there has been a substantial increase in the number of genetic polymorphisms identified by GWAS and meta-analyses to be associated with osteoporosis. The majority of investigations on the selected SNPs associated with a low bone mass were based on adults rather than adolescents [41]; therefore, there is a lack of a good reference for the selection of SNP candidates in the present study. In addition, very few studies have looked into the association between the identified SNPs with bone quality in AIS patients. Polymorphisms associated with osteoporosis such as those in osteoprotegerin (OPG) and interleukin-6 (IL-6) were found to be associated only with lumber spinal BMD in cohorts with a relatively small sample size of AIS patients [42,43]. In this study that investigated the Hong Kong adolescent population, we compared genotype frequencies in the healthy controls and AIS participants; however, no significant difference was observed in the genotype frequencies of the studied polymorphisms between the two groups. A combined analysis of the AIS and control groups provided evidence indicating the potential interaction between *WNT16* and Vit-D pathway genes (*VDR* and *VDBP*) on the serum 25(OH)Vit-D level and bone quality parameters. The serum 25(OH)Vit-D level was reported to be positively correlated with bone qualities in adolescents [17]. Studies on the genetic regulation of 25(OH)Vit-D levels have identified Vit-D metabolism-related genes such as *VDBP*, *VDR*, *CYP2R1*, *CYP24A1*, and *DHCR1* [44]. Large-scale GWAS studies [45,46] suggested that *VDBP* rs2282679 is a significant locus that regulates serum 25(OH)Vit-D levels. The risk of Vit-D deficiency was found to increase by approximately 50% for each additional copy of the minor C allele of rs2282679 [46,47,48]. Our findings further suggest the presence of multi-locus interactions among *WNT16* rs3801387, *VDBP* rs2282679, and *VDR* rs2228570 on the serum 25(OH)Vit-D level and trabecular area in the AIS and control groups.

A meta-analysis on GWAS data confirmed that *WNT16* variants are associated with a risk of low-energy trauma fractures [23]. Many candidate gene association studies provided evidence that allelic variants in and around the *WNT16* locus are strongly associated with BMD at several different skeletal sites and hip fractures, supporting the role of *WNT16* in determining the risk of osteoporotic fractures [49]. Another genome-wide analysis associated missense SNPs at the *WNT16* locus with total-body BMD variability in 2660 children of different ethnicities and with peak bone mass in premenopausal women identified [50]. Collectively, *WNT16* appears to have a positive effect on BMD and bone strength, while mutations in *WNT16* produce a systemic low BMD phenotype. Given that the identified SNPs may not be the causal variants, and that there are potential confounders affecting bone qualities and serum 25(OH)Vit-D levels in our clinical cohort, an animal study on *Wnt16* global knockout mice was used to determine if *Wnt16* interferes with Vit-D status and bone quality parameters when the mice are given different levels of dietary Vit-D. Although mice are different from human in terms of skeleton growth rate, studies on bone growth in mice with different genetic backgrounds have demonstrated a linear and rapid increase in cortical bone accrual from week 2 up to the age of 8 weeks [51]; this reflects a rapid periosteal and endosteal expansion that can, to a certain extent, act as a model for studying the effects of *Wnt16* and Vit-D diet in the pubertal period. In this study, we observed a significant decrease in the serum 25(OH)Vit-D concentration in the Vit-D deficiency group in both WT and *Wnt16* global KO mice. The results from a previous study using a similar Vit-D dietary treatment on mice and measuring serum 25(OH)Vit-D concentrations are comparable to our observations, confirming the effect of Vit-D deficiency in reducing circulating Vit-D levels in mice [52]. In addition, 10-week-old mice demonstrated significantly lower serum 25(OH)Vit-D concentrations in the absence of *Wnt16* under the same dietary conditions, suggesting that *Wnt16* may influence Vit-D metabolism in vivo. The expression of *Wnt16* in different tissues is not clear. A previous study showed that conditional knockout of *Wnt16* in osteoblast lineage cells did not affect the weights of the majority of internal tissues, including the kidneys, liver, spleen, and fat. Intriguingly, *Wnt16* global knockout significantly increased *Vdbp* mRNA expression in the liver where most of the VDBP protein is synthesized [53]. While the biological effect and underlying mechanism of *Wnt16* on VDBP expression and Vit-D status and metabolism require further investigation, our present findings provide a clue of a new biological function for Wnt16 on bone quality through interactions with Vit-D metabolism.

Vit-D is an important factor in skeletal growth, regulating calcium–phosphate homeostasis, and bone matrix mineralization. Vit-D deficiency during the rapid growth period was associated with inadequate skeletal mineralization, which leads to rickets and even osteoporosis. We previously reported a high prevalence of Vit-D insufficiency and deficiency (64% and 11%, respectively) in Hong Kong adolescents, and the circulating Vit-D level was significantly correlated with bone density and bone quality parameters [17]. Considering the higher prevalence of AIS in areas located at high northern latitudes, Vit-D status has been speculated to be associated with the risk of curve progression in AIS [40]. In this study, serum 25(OH)Vit-D level was found to be slightly higher in the AIS group when compared with the controls. According to the reported negative correlation between body weight and serum 25(OH)Vit-D levels [40,54], this discrepancy could be attributed to the lower body weight in the AIS group [1,39,40]. On the contrary, it was also reported that AIS patients had lower serum 25(OH)Vit-D levels and lower body mass index (BMI) compared with the control subjects, [55,56] and whether the low BMI was associated with curve progression remains controversial [57,58]. Recently, a mendelian randomization study suggested a potential causal effect of BMD on the incidence of AIS, which was found dependent on BMI, body fat mass, and Vit-D levels [41]. While further longitudinal studies are warranted, researchers have attempted to explore the effectiveness of Vit-D supplementation on bone quality and curve progression in AIS patients [59].

Vit-D supplementation with adequate calcium intake and sun exposure is widely used for improving bone health in children and adolescents with Vit-D deficiency or insufficiency [14,34]. However, clinical studies have not provided concrete evidence to support this practice. Brustad et al. performed a double-blinded, randomized trial involving 623 Caucasian pregnant mothers and their 584 children. Vit-D supplementation at 2800 IU/day (high-dose) vs. 400 IU/day (standard-dose) daily from pregnancy week 24 until 1 week after birth resulted in serum 25(OH)Vit-D levels of 42.59 ± 14.30 ng/mL and 29.29 ± 12.66 ng/mL, respectively. The high-dose group had a higher whole-body BMC according to DXA scans at age 3 and 6 years old [60]. Rosendahl et al. conducted an RCT involving 975 healthy term infants. The infants were given a daily oral Vit-D supplementation of 400 IU or 1200 IU from age 2 weeks to 24 months, resulting in serum 25(OH)Vit-D levels of 34.70 ± 7.85 ng/mL in the 400 IU group and 47.16 ± 10.46 ng/mL in the 1200 IU group. No differences were found between groups in terms of bone strength measures at 24 months, including bone mineral content, BMD, and bone cross-sectional area [61]. These two recently published papers suggested that the optimal dosage of Vit-D supplementation for good adolescent bone health has yet to be determined. Various factors such as inconsistent basal serum 25(OH)Vit-D levels, treatment duration, daily amount of Vit-D supplemented, unsatisfactory drug compliance, abnormal Vit-D metabolism, and even extrarenal Vit-D activation may lead to inconsistent findings [62,63]. Our findings further suggest that adolescents with genetic variants in *WNT16* may require a higher daily Vit-D intake, but this requires further investigation.

Our findings from the WT mice indicated that higher cortical bone accrual could be achieved by a sufficient Vit-D dietary intake of 1000 IU/Kg. Diets with a Vit-D deficiency or high dosage of Vit-D would lead to unsatisfactory cortical bone accrual. Furthermore, the Vit-D deficiency treatment group showed a significant reduction only in cortical bone at the age of 10 weeks, suggesting that a duration of at least 6 weeks is required for the effect to be detectable. A previous animal study using 20,000 IU per kg of dietary Vit-D on female mice observed no significant change in cortical bone mass in the tibia bone from weaning up to 8 weeks of age [64], yet our findings suggest that by the age of 10 weeks, there should be significant reduction in cortical bone quality with 20,000 IU/kg Vit-D. A recent clinical study found that radial and tibial BMD was significantly lower with a daily dose of 4000 or 10,000 IU compared with 400 IU per day for 3 years in healthy adults, indicating high-dose Vit-D may not have beneficial effects on bone mineral density [65]. This supports our observation of lower cortical bone quality in the high-dose Vit-D group in mice undergoing rapid growth. However, in the absence of *Wnt16* along with a high dosage of Vit-D, there was no significant reduction in cortical thickness or density in the corresponding WT group, suggesting a potential crosstalk between Vit-D and *Wnt16* in the context of cortical bone accrual. This exploratory study examined the combined effect of ablating *Wnt16* and different dosages of dietary Vit-D on cortical and trabecular bone qualities during the rapid growth phase. These findings may shed light on the novel role of Vit-D in regulating cortical bone quality in adolescents via the WNT16 metabolic pathway.

This study has its limitations. First, a larger sample cohort including wider spectrum of scoliosis severity is warranted to validate the interaction between *WNT16* SNPs and Vit-D-related SNPs on the benefits of vitamin D supplementation for future clinical use. Second, only three SNPs were selected, while other genetic variants were not evaluated. Third, the effect of *Wnt16* overexpression on Vit-D supplementation outcomes was not investigated. Fourth, the present study focused on bone qualities in AIS, and the risk of other extra-skeletal conditions that were related to Vit-D level and vitamin D supplementation was not investigated [66].

In conclusion, *WNT16* may regulate the serum 25(OH)Vit-D level and bone quality, which might be associated with *VDBP* expression. Further investigations with a larger sample size and wider spectrum of scoliosis severity are required to validate our findings regarding the interaction between *WNT16* and Vit-D status in patients with AIS.

## Figures and Tables

**Table 1:** Demographic, environmental, and clinical factors of the recruited subjects.

	AIS (*n* = 318)	Controls (*n* = 201)
Age and stage of puberty		
Age (years)	14.02 (13.03–15.24) **	13.47 (12.88–14.52)
Maximum Cobb angle (°)	27 (22–35)	NA
Breast stage	3 (3–4)	3 (3–4)
Pubic hair stage	3 (2–4) *	3 (2–3)
Anthropometry		
Body weight (kg)	43.89 (39.40–48.03) **	47.20 (41.40–53.00)
Body height (cm)	155.85 (130.00–175.50)	156.20 (136.00–170.00)
Sitting height (cm)	83.00 (80.50–85.00) *	83.50 (81.25–86.15)
Arm span (cm)	156.00 (151.00–161.40)	155.00 (149.70–159.50)
Vitamin D status		
Serum 25(OH)Vit-D (nmol/L)	50.10 (17.40–126.00) **	40.50 (15.00–90.00)
Vit-D sufficient	137 (50.2) **	56 (27.9)
Vit-D insufficient	120 (44.0) **	130 (64.7)
Vit-D deficient	16 (5.9)	15 (7.5)
Bone parameters		
Left FN aBMD (g/cm^2^)	0.68 (0.63–0.74) **	0.78 (0.70–0.89)
Right FN aBMD (g/cm^2^)	0.68 (0.63–0.75) **	0.78 (0.71–0.89)
Z-score of Left FN aBMD	−0.55 (−0.98–−0.12) **	0.10 (−0.67–0.97)
Z-score of Right FN aBMD	−0.54 (−0.92–−0.16) **	0.03 (−0.64–1.03)
Total vBMD (mg/mm^3^)	269.40 (228.20–319.10) *	287.30 (232.50–343.85)
Total bone area (mm^2^)	183.20 (109.30–275.10)	183.00 (110.20–264.50)
Cortical vBMD (mg/mm^3^)	749.80 (665.50–811.80)	759.00 (668.75–821.70)
Cortical thickness (mm)	0.59 (0.37–0.81)	0.66 (0.38–0.86)
Cortical area (mm^2^)	33.70 (20.10–44.70)	35.60 (20.75–47.25)
Cortical bone perimeter (mm)	55.10 (42.40–69.80)	55.20 (41.90–68.10)
Trabecular vBMD (mg/mm^3^)	138.50 (76.50–219.70) **	153.40 (72.20–242.80)
BV/TV	0.12(0.10–0.13) **	0.13(0.11–0.14)
Trabecular number (mm^−1^)	1.64 (0.69–2.21) **	1.72 (0.91–2.45)
Trabecular thickness (mm)	0.07 (0.07–0.08) *	0.07 (0.07–0.08)
Trabecular area (mm^2^)	143.80 (125.00–163.55)	143.9 (121.30–162.15)
Trabecular separation (mm)	0.54 (0.49–0.61) **	0.50 (0.45–0.58)

AIS, adolescent idiopathic scoliosis; FN, femoral neck; aBMD, areal bone mineral density; vBMD, volumetric bone mineral density; BV/TV, trabecular bone volume to tissue volume ratio; NA, not applicable. Results expressed in median (range) or number (percentage). * *p* < 0.05. ** *p* < 0.001.

**Table 2:** Multi-locus interaction models in GMDR analysis.

	Number of Loci	SNP Combination	CVC	TA (%)	*p* ^1^
Serum 25(OH)Vit-D					
	1	*VDBP_rs2282679*	10	60.36	0.001
	2	*WNT16_rs3801387, VDBP_rs2282679*	10	59.64	0.003
	3	*WNT16_rs3801387, VDBP_rs2282679, VDR_rs2228570*	10	60.08	0.006
Total vBMD					
	1	*VDR_rs2228570*	6	51.58	0.355
	2	*VDBP_rs2282679, VDR_rs2228570*	10	58.68	0.01
	3	*WNT16_rs3801387, VDBP_rs2282679, VDR_rs2228570*	10	51.39	0.381
Total bone area					
	1	*VDR_rs2228570*	10	53.23	0.189
	2	*VDBP_rs2282679, VDR_rs2228570*	10	56.7	0.041
	3	*WNT16_rs3801387, VDBP_rs2282679, VDR_rs2228570*	10	55.91	0.074
Cortical bone perimeter					
	1	*VDR_rs2228570*	10	54.58	0.103
	2	*VDBP_rs2282679, VDR_rs2228570*	10	57.24	0.029
	3	*WNT16_rs3801387, VDBP_rs2282679, VDR_rs2228570*	10	54.88	0.117
Trabecular area					
	1	*VDR_rs2228570*	8	52.68	0.239
	2	*VDBP_rs2282679, VDR_rs2228570*	10	59.62	0.004
	3	*WNT16_rs3801387, VDBP_rs2282679, VDR_rs2228570*	10	56.73	0.044

CVC, cross-validation consistency; SNP, single-nucleotide polymorphism; TA, testing accuracy. ^1^ Based on 5000 permutations, fitting age, and AIS diagnosis as covariates.

## Data Availability

The data that support the findings of this study are available on reasonable request from the corresponding author.

## Supplementary Materials

The following supporting information can be downloaded at: https://www.mdpi.com/article/10.3390/biomedicines12010250/s1, Table S1: Allele frequencies of the selected SNPs in the clinical cohort; Table S2: Associations between clinical phenotypes and selected SNPs in the clinical cohort.
