# Supplementary material for: Potential Interaction between WNT16 and Vitamin D on Bone Qualities in Adolescent Idiopathic Scoliosis Patients and Healthy Controls
Source: Biomedicines. 2024 Jan 22;12(1):250. doi: 10.3390/biomedicines12010250 (PMC10813331; doi:10.3390/biomedicines12010250)
Supplement: Supplementary file 1 [file biomedicines-12-00250-s001.zip › biomedicines-2673883-supplementary.pdf]

Supplementary Table S1. Allele frequencies of the selected SNPs in the clinical cohort

SNP	Gene	Position	Cytogenetic Band	Nature of SNP	Major Allele	Minor Allele	MAF of CHS from 1000 Genomes	MAF in Our Genetic Study	
								AIS Patients	Controls
**rs3801387**	**Wnt16**	**7:121334711**	**7q31.31**	**Intron**	**A**	**G**	0.152	0.124	0.139
rs2282679	VDBP	4:71742666	4q13.3	Intron	T	G	0.319	0.253	0.269
rs2228570	VDR	12:47879112	12q13.11	start lost	G	A	0.386	0.451	0.470

MAF, minor allele frequency; CHS, Southern Han Chinese. All genotyped SNPs of all subjects in two cohorts were in Hardy–Weinberg equilibrium with $p > 0.05$.

Supplementary Table S2. Associations between clinical phenotypes and selected SNPs in the clinical cohort

Clinical Phenotypes	*Wnt16*_rs3801387 (A/G)		*VDBP*_rs2282679 (A/C)		*VDR*_rs2228570 (G/A)	
	p-Value	OR (95% CI)* / β (95% CI)	*p*-Value	OR (95% CI) */β (95% CI)	*p*-Value	OR (95% CI) */β (95% CI)
AIS diagnosis [a]	0.337	0.832 (0.571–1.211) *	0.504	0.905 (0.675–1.213) *	0.510	0.916 (0.705–1.190) *
Bone parameters						
Left FN aBMD [b]	0.989	0.000 (−0.017–0.017)	0.579	0.004 (−0.010–0.017)	0.743	−0.002 (−0.014–0.010)
Right FN aBMD [b]	0.974	0.000 (−0.017–0.017)	0.653	0.003 (−0.010–0.016)	0.664	−0.003 (−0.014–0.009)
Z-score of Left FN aBMD [c]	0.403	0.066 (−0.089–0.222)	0.863	−0.011 (−0.132–0.110)	0.769	−0.016 (−0.124–0.091)
Z-score of Right FN aBMD [c]	0.450	0.060 (−0.096–0.216)	0.896	0.008 (−0.113–0.129)	0.948	0.004 (−0.104–0.111)
Total vBMD (mg/mm^3) [b]	0.182	7.006 (−3.303–17.315)	0.968	−0.163 (−8.260–7.934)	0.510	2.411 (−4.777–9.598)
Total bone area (mm^2) [b]	0.293	−2.576 (−7.383–2.231)	0.257	2.171 (−1.592–5.934)	0.243	−1.992 (−5.341–1.356)
Cortical vBMD (mg/mm^3) [b]	0.191	7.881 (−3.933–19.695)	0.678	1.961 (−7.324–11.246)	0.308	4.283 (−3.957–12.522)
Cortical thickness (mm) [b]	0.274	0.021 (−0.017–0.058)	0.751	0.005 (−0.025–0.034)	0.486	0.009 (−0.017–0.036)
Cortical area (mm^2) [b]	0.330	0.941 (−0.956–2.838)	0.414	0.621 (−0.870–2.113)	0.797	0.174 (−1.150–1.497)
Cortical bone perimeter (mm) [b]	0.392	−0.322 (−1.061–0.417)	0.212	0.369 (−0.211–0.949)	0.225	−0.317 (−0.831–0.196)
Trabecular vBMD (mg/mm^3) [b]	0.384	2.246 (−2.820–7.312)	0.797	0.524 (−3.467–4.514)	0.919	−0.182 (−3.700–3.337)
BV/TV [b]	0.389	0.002 (−0.002–0.006)	0.802	0.000 (−0.003–0.004)	0.915	0.000 (−0.003–0.003)
Trabecular Number (mm^{-1}) [b]	0.410	0.019 (−0.026–0.063)	0.513	0.012 (−0.023–0.046)	0.685	−0.006 (−0.037–0.025)
Trabecular Thickness (mm) [b]	0.889	0.000 (−0.002–0.002)	0.725	0.000 (−0.002–0.001)	0.821	0.000 (−0.001–0.001)
Trabecular Area (mm^2) [b]	0.282	−2.831 (−7.993–2.331)	0.455	1.541 (−2.506–5.588)	0.368	−1.649 (−5.247–1.949)
Trabecular Separation (mm) [b]	0.488	−0.006 (−0.025–0.012)	0.516	−0.005 (−0.019–0.010)	0.706	0.002 (−0.010–0.015)
Serum 25(OH)Vit-D level [d]	0.039	3.635 (0.180–7.089)	**<0.001**	**−4.844 (−7.521–−2.167)**	0.392	1.046 (−1.355–3.448)

AIS, adolescent idiopathic scoliosis; FN, femoral neck; aBMD, areal bone mineral density; vBMD, volumetric bone mineral density; BV/TV, trabecular bone volume to tissue volume ratio; Vit-D; vitamin D; OR, odds ratio; CI, confidence interval. $p < 0.0167$ regarded as significant after Bonferroni correction. a, analyzed by binary logistic regression, fitting age as covariates. b, analyzed by linear regression, fitting age, AIS diagnosis, arm span, body weight, breast stage, and pubic hair stage as covariates. c, analyzed by linear regression, fitting AIS diagnosis, arm span, body weight, breast stage, and pubic hair stage as covariates. d, analyzed by linear regression, fitting age, and AIS diagnosis as covariates.
